# Supplementary material for: Identification and Fine Mapping of the Candidate Gene Controlling Multi-Inflorescence in Brassica napus
Source: Int J Mol Sci. 2022 Jun 29;23(13):7244. doi: 10.3390/ijms23137244 (PMC9266383; doi:10.3390/ijms23137244)
Supplement: Supplementary file 1 [file ijms-23-07244-s001.zip › Supplementary File S1.pdf]

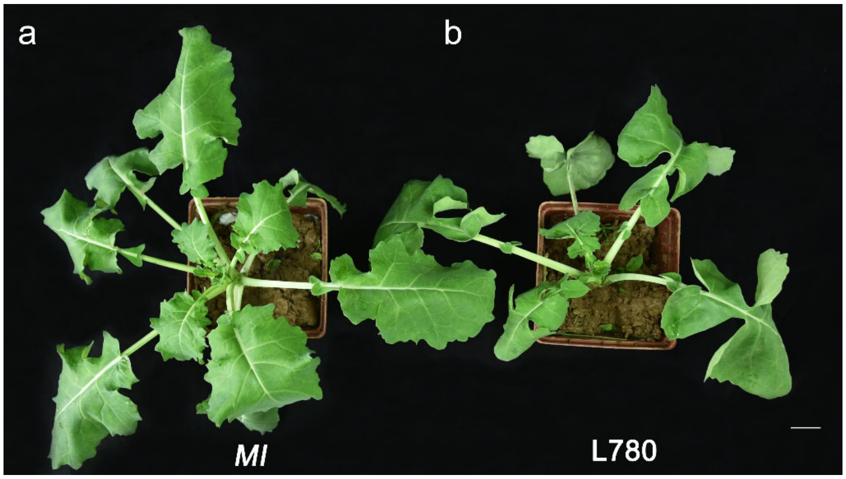

Figure S1. Seedlings plant of *MI* mutant and wild-type L780. (**a**) the seedlings plant of *MI* (**b**) the seedlings plant of L780; bar = 2 cm.

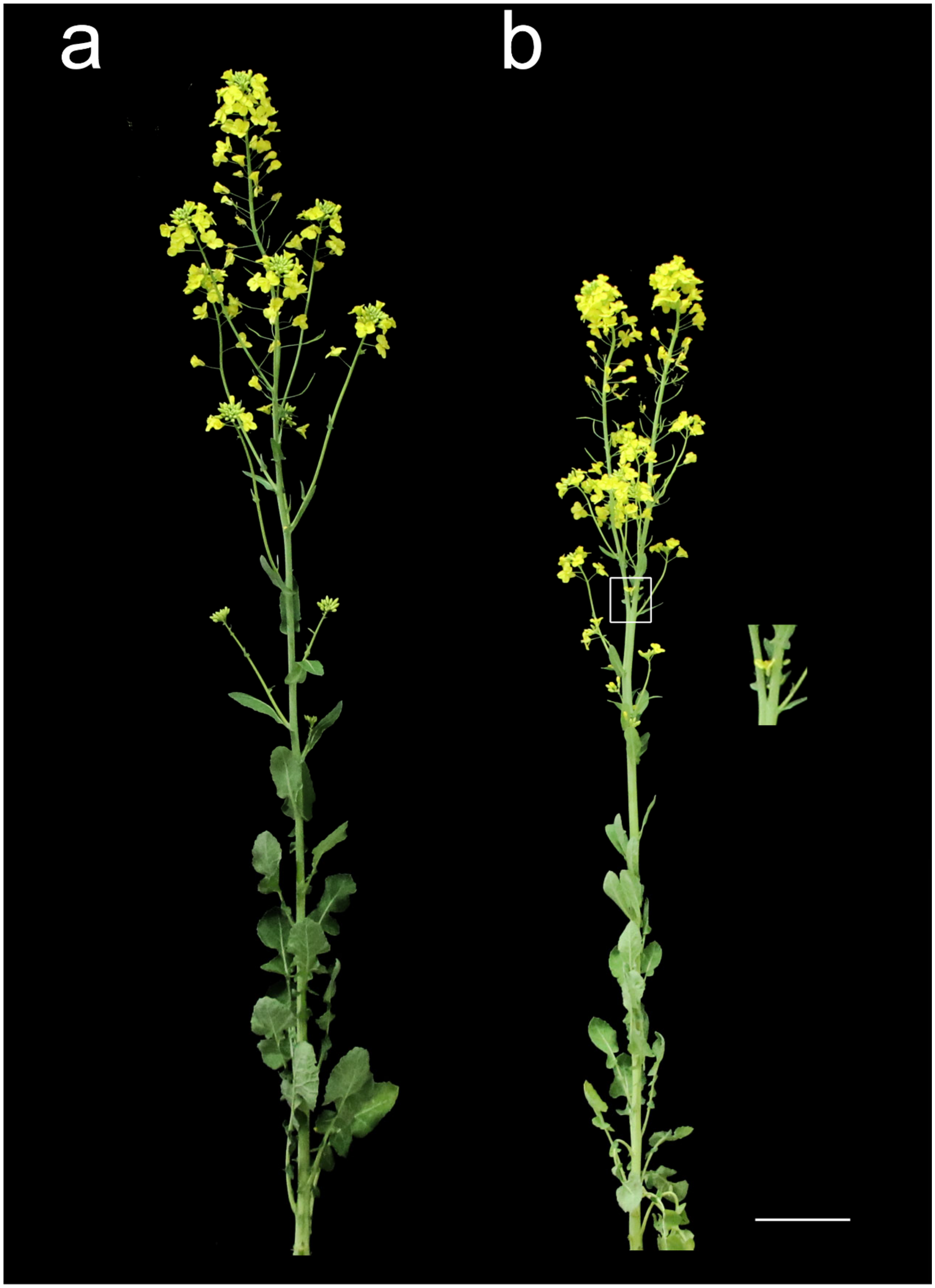

Figure S2. Multi inflorescence-related traits of *MI* at flowering stage. (**a**, **b**) Plant morphology of L780(**a**) and *MI* (**b**); bar = 10 cm; the white frame indicates the enlarged part.

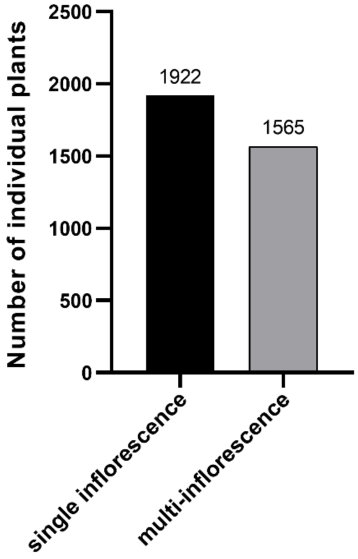

Figure S3. Frequency distribution on multi-inflorescence traits in BC_4F_1 population. The number above the column represents the actual number of individual plants of multi-inflorescence and single inflorescence respectively.
