# Supplementary material for: Identification and Fine Mapping of the Candidate Gene Controlling Multi-Inflorescence in Brassica napus
Source: Int J Mol Sci. 2022 Jun 29;23(13):7244. doi: 10.3390/ijms23137244 (PMC9266383; doi:10.3390/ijms23137244)
Supplement: Supplementary file 1 [file ijms-23-07244-s001.zip › Supplementary File S2.pdf]

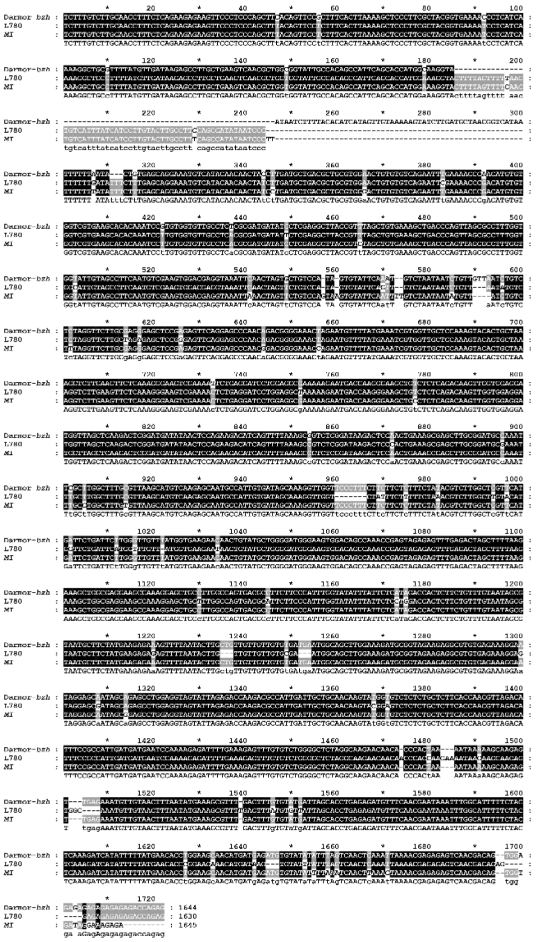

Figure S4. The promoter region sequence alignment of *BnaA05g08870D*. The chart contains 4 sequences, the gene sequence of Darmor-*bzh*(as a reference), the gene sequence of L780 and *MI* , the consensus line. Spaces indicate insertion or deletion and common bases are in black.

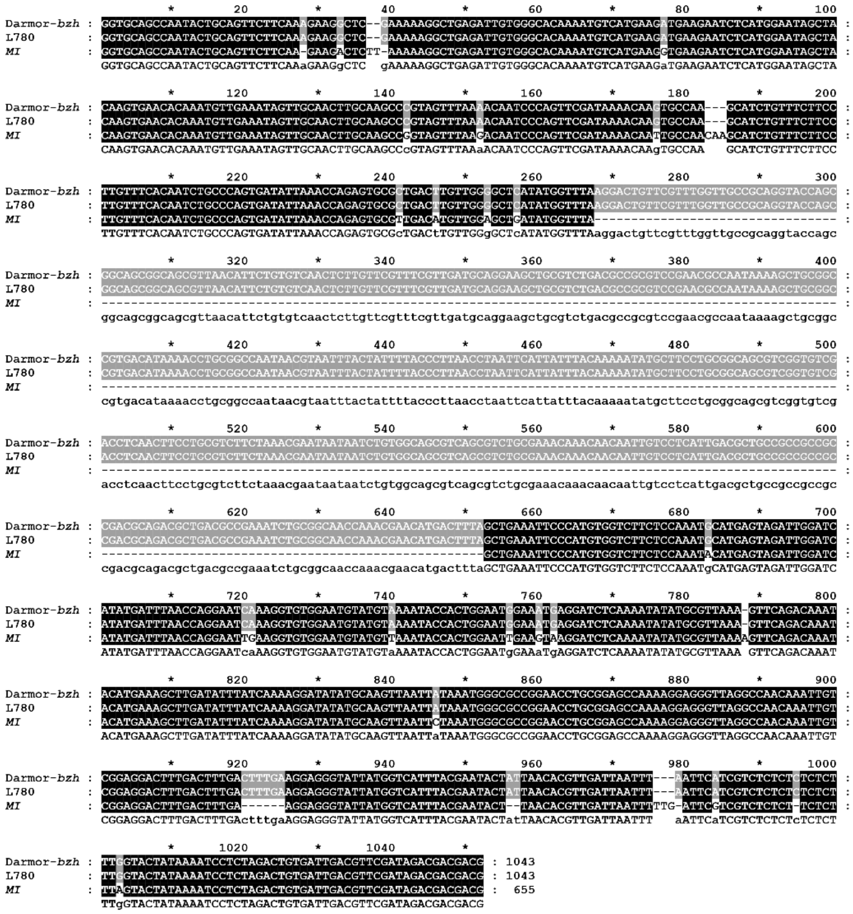

Figure S5. The promoter region sequence alignment of *BnaA05g08890D*. The chart contains 4 sequences, the gene sequence of Darmor-*bzh*(as a reference), the gene sequence of L780 and *MI* , the consensus line. Spaces indicate insertion or deletion and common bases are in black.

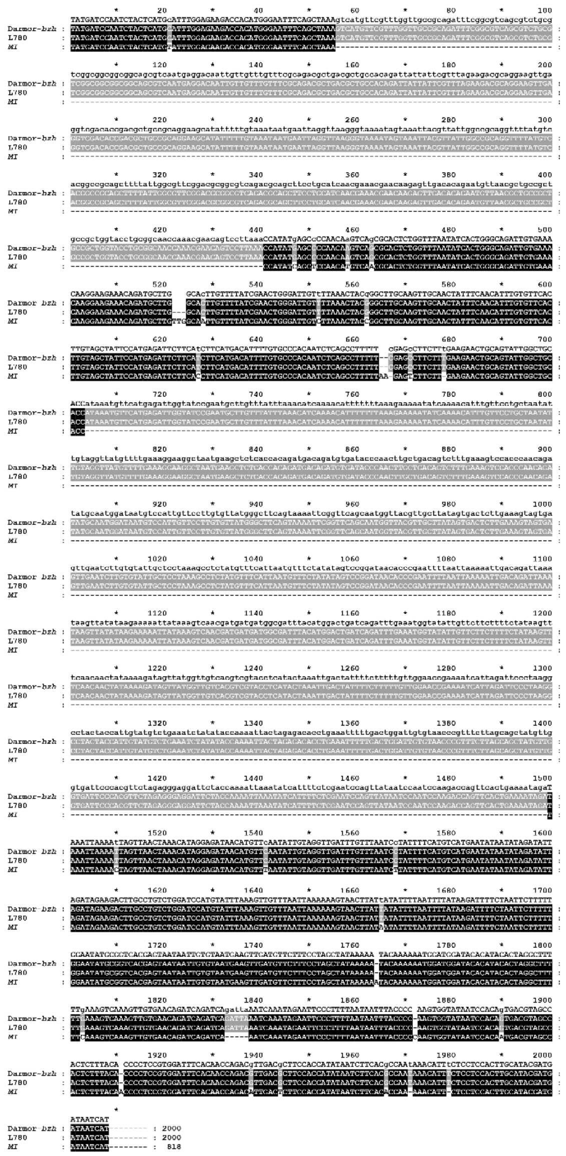

Figure S6. The promoter region sequence alignment of *BnaA05g08900D*. The chart contains 4 sequences, the gene sequence of Darmor-*bzh*(as a reference), the gene sequence of L780 and *MI* , the consensus line. Spaces indicate insertion or deletion and common bases are in black. The complete sequence of large fragment insertion was not acquired due to the Poly structure and the obtained sequence is listed as below.

>large fragment insertion

CCCATTTTCCTACAAAATAAACTCCTCTAGAATTCATTAAACATGATCAACATAAACTTTCCCCTAACGATATAATAGACCCTTTTAAGTAGGGGATGATGGTTCAAACTCATACCTGATAGACGAATAGTTAGGGGAAGATGGTCGGAGCTTGTCT

CAGTGATTCACAATGTTTACTTTCAAAACTAGTTGAAAACAGAGAGGTGATAGCAACTTATATGGAGATCTATGTTCTTACCTACTTAAGAAGGAGTGGTATTGGCTCCACGGGAACAGGAAAAAGCATGTGCGCACATGTTATATTACAGGAAA

TAATGTGACGCCCCTCATCTCTGCTCACAATTACGTAAAATGATCGACTGGTTACCATATAGTTTATTGGAACCCATCCGATTAATGAAAGAACATATTATTTATAACGGCTAACGTGGACCCCAAATACATATGTTAAAGCAGCTGCTATGGTACCT

TTTTTTTTGTCAACCCAGCTATTGTAGCTTAATTACACAATGTAACCATAACCTAAAATATTAGAGTATCCTAGCTAAATTACCCAATGTAACCATAAGCTTATTTTTTTTTTTAAAGCATAACCATAAGCTATAATATTACAGAATTTACCATTAAGT

TCCTCACTTTAGGGTATATATTTAAGGAACAAAAGGTTAACAGTAGAGAAGATAAGGATGACAATTACATTACCCAAAGTTAAAAAATATTGTTTTTCCTCCACCGCTTATTTCCGAACTTCGATGACGTACATCTTGTTTCCACCACCTGCTTATT

ATGGATTACTATTCCAGATATTTACTAAGCTTAGCTTACAAAAGAAGACTTATAGATGATAGAATTTATAGACAGTGACAGAAATAAATAAAATAACAATGACCTGTTTTGTCATGTCAGAGAAAGTCGCATCATGATGGTTAACTAGCCCTATATA

GGCTGCGGCATTAAATGCTAAGATATGAGAAAGATTTGGTCCAATAACCTCCAACTGGAGGTGAAACCGGTTTCTACAGCATGTTGAAGTCTTTATATAGATGCTTCCCCTATTAACTTTTACAACACAACTTACCTTTACTGAGACCGGATAGGC

TCTGAGAAAGCAGATATTAACTTCAGTTAACTGGTAGTCCGAGGAGTTCATAAGATTCGAAAAGGTGATTCCTTTATACTTGAAGACCATAACTATTTCTATTTCTTACTTACGAATGGGACCCAAATACGTATCATCTCGGACTAATGTTCTTCTTT

TATATGGACAGATGGCACGTTTGGTTCGTGTTTTTAAGGGACAGTGGTTCAAGTCGCAGGATGGTGTGTGGAGGTTCGACCAAGATCCTACCATGACAGGGCGTGACATACTCATTGCAAACACCGAGCACCTTGAAACACTTAAAGACCTTGT

CAGGGCTGTCTTCTGTCTTCGTACAGAAACCCCGATGGTAGTTACTTTCCAGCTCCCACAGTGGATGCTCGAGCCTGATGGCGGGACGTGGCCTCCACATAACCTAAACAGTAACGCAGACGTCGATATGATGATGAGCGTACATGACTGGAAT

GTAGAACCAAGGCTTTGCGTCATATTTGGTGCTCAGGATGTGGCTACATACCAGTTCAGGTGCAGAAGACCTTTTACCATCGGATCTTCCACATTCCTAGCCCAAGGTGTGACGGAGGAGCAACACATGGCGCGTGTGTTAGGTATATTTACGTA

CACATGAATATCGTTTGCAATACATAATAAAAAATGATTCTAAACCATTACCAACATCTTTGTTGATAGATATGATACGTGGTAACGAGCTACTGTGCAGTGAGCAAGTGCTTAACGAAATCTTTGACGAAGAAAAAATGGTCTTACTCTATCGTTT

CTCTTTGGAGATTCAAAAAGCCAAAAACAGTCTCGACCTCAATTTAGGACCCATGGTGGAAACTGATGACCACATTGTACCTAATATCGGTGGTGAGGGGGGGACACATGTTCAGCATGGAGGTGGAGTTCCCAACACTGAGGTTCAACCAGA

CGTAGCAAGGGGTGAGCTTCCAACCGGTGACGTTCCCTTTAATACTCCTCCACATTTTGGTATGTTTCTCTACTTACATTTTGTATTTAACAATTAACTATAATAATTATAACGATATATATTAGAGTAAACGGTTGCCTATAGCTTATTTGTTTAGATA

CTTAACATATATTACAAACCTAATAGTTCAACGGCTTCCATTTGTAGGATATCTACCATTCAACACAGCTGCATCCATGACGACCACTCCTCGTCAGAGCATGTATGGGCCACACTATTATACGCCGCATATGGGAAGCATGGCTGTTAGAGGAAGC

TATTGGGAGAACTTGATGTCATCCAGGTATGCAGTTGATGTGCAGAGAATTTATGGAGTACCAGGATCTGAATACGTTGGATATTCCCCCAATGACCTCAATATCGGTAACCCTACTGGTCCACCTATGCTTGGGTCCCCAAATGGTCCACCTATGC

ATGGGTACGGGGTTCCTATTGAAGTTTCTTCTACGGCTTCATCTACGGAGGTGCATAACGTTCGTGACATTGGAGTAACAGTCAATACATCCAAAGAAACCGGTATCAGAGATGGCATCTGTGTTGAGAAAGGTATGAGTAGACTATTTTCAAAA

TTATGGAAATTTACAATCCCATAATTACTTCGTAACAGAGCATAGCAACTAAAAGTCAATTACTTATATCCTGAACTACATTTTCTTTATTTTTTTCCACTGAAAGGGGAGTCCTCCAATCGGCCAGAGCCTGCAACTCATGCGGACTTGGACGTTA

ACTTCCAGATTGGAGACACAACGCCGGCAGCCGATGTTGGTGTTGATGCTGGAGAGGGTCGGGACGCTTGAAGAATCTGCCAAACAAGATGTAAAATAAATAAGGGACAACTTTATGTATAATGTCTAAAACCTTTTGGCGTTGTGAAATATGC

TCGTAGACTTGGTATGCATGGTAAAATATGGGAGTTCGTTTACTATGATGACGTTTTCTCGAGCCTCCAGTCGGTGTAATAGTTTGTCTTCTATGAAGATTGTATGTGTGATATTAATTTTAGTATGTTGCCTTTGTAAAATACTTTTGTTTCAATGATT

ATATAACGTAAACGTAAACCGCAATTATATTCTTATTTTGGTTATTCAACAAAATAACCCATAAACTTAAACCGGCTTATATAGGTAAAAAATTGTTGTCTAAAATTACTCCCATCACTAAAATGATGTGTGTATTGCCACCTTTTCGAACTTAGTTTT

CCAGTTAGTAAACGATATGGAAGGAATCTTAACAACTATTGCTTTCATCAAAGTTGAGTCTTCCATCGTTACATTAAAGACAATGTTGTTTAAGTTAGCCGTAACCTATTGTCTCTCTTCATAATAATAGTACAACAACTATGAGAAGAAAGTCAAA

TTACATAGTTCCCCATGAAATGAACTTCTCATATTTTGTACCCTGTAATGCTTCCAACTCTCATCTAATAACTAAATCACACAAAGAGCCATATGCAACAAGAGGGGAAAAAAAGAGTTTAACAGCGAATCTTATAATATGACACATTGTTCCTCAA

ACAAATAGAATAGACAAGGTTGACATAAGACATGACGTAATAACAGGTACGCTTCGAGGAGAACCGGTACCGTCTTCTACCAAAGGACTTTATTAAACCGGGTAAAAAAGCGTTTCCTACAGCTTCTGATGAAATTCATACACCATAATCGCTGC

CTTTTGTGCTTCCACTGGAATGACAGTTGGAGTTATAGACCTACAGCCGTCAAATCCAAACAGCGCATGTGCTTGGATCAAAAGACAGGCGGTCAACGCCGCATCTGCGGGATTAGTGTTTTGAGCAACACCTTTGATTCTTTCAACCGCAAGT

TTGTTGCCGATAGTAGGAACACCATTACCTAAACGTCTCACTATATAAGGAAACATGTCAGAGATTGGTAGCAGCTCTTTTACCAACGCTTCATCCGTAATAAGACCTTGGTTGCAGTCCAGAACATATATCTTCGACGAAGGGATATCTACACAA

AGGCCAACCCAATGTCTGCGATGGACGTTGAATGGAATATACCACCTCGCCACAGCAATGTGTCTCGGGGAACTCTTTCTAACACAATCTACCACTCCCTTACTGAAAACATATTTTTCCCTCTGCTTTGACTTTTTAAACTTTGGATAATTCCTTC

CAAGCAAAATCTGCACACGACTGTCAAGGAACACTGGAATGGTATCGATCTTACCCAACGCTTCGTTGTTAACGTTGAATGCAACAACCCTTGTAAGTATATCTATGACCTGAGTAAATATAATAAAGCATCGGTGAGGATACAAATTAGACGGT

AATAAATAGTCAATTACGCGTAACGTACTCGCCCAGGAAGAAATCTGTTCCTCTCTCCGATGTCGACCATGTCCTTCCCAGTTACTGACAAGCCCGCAACATTGATAACACTGGAAAGAAATACAAAAGTATGAGTAAGTAGAGTCCCTACCCTG

GTAAATTTCAAGAAGAAAACAATCAAATATATTATTCTTCAAAATAATGAATAGATTTTTGTAAAATACCACTCTTTCTTAAGTAAGAGCTTCAGACGCAGGTACTTATCACGAATCCCAGAACATGCATACCCAATCATCCCTAGAAGTTGTCCTT

CACGAGCCCTATTAACAATTGGGGTACCGCACTGGTAGTCGGTGATAAGTTGCGGTGGAACATGCCGCCTCCGCTTGCTTTTACGGCAAGTCAGAGTTTCTTCATTATCGCAAACCACTGCTTCAATAGGTTCTTGAACGGAGATATTGTTATTAT

GTCCCATGTCGCCAGCTTCCTGCTGCCGTTCAATTCCAAGCTCCTGAGTTAGGCCAAGTGAGAAAGAAGGAATCTCATGTCCAAGAAACACGTCGACCGCCTACACACATTTAACATCTTTGGCTTTCGTAAAAATAGCGTACATAGTGAACAA

TCAAAGAATTTTAAAAAATTGTTACATTTACTCCTCCCTGTTGGGCCGTCTGGCTGACACCACACAAAGATGCATGCTGATTTATACCACCGTCAGCGTTCCCAGCGGAACGGACCTTCCGAGGGAAGGAGATAAGGTAGGTCGCAGATCCCTAT

AATTTTGAAAAAAAATTTGAAGACGGAAAAGTATTAAATGCCTATTTTTTAAACTTGCTTCTACATTTTCACAAGCTTCAGAAGTGGTATGGAGGGAAACATTATAACCGAATACTAATATGGTAAACAAAGTAGTGTTGAATTGTAAAAATATAT

GAAACAGAGACGTTACTTCGGATAGTTCCTTTGAAAGCTGTTTCGCGGACATACGCTTCTTTGAATGAATACTGGATGTTTCATTTTTCCGTTGACTATCTTCAGGATGCCCGCCGGCAGATAAGGGGGATCGCTGCCTATGGGACTCCGCCTCAG

ATCCCTGACACAATCATCTAACTTATAAGGAGTTCTCTTCATACTTAAAATAACAAAAATTTCTTCAAAATTATATGTCACCAAAGGACGCTACTTTGAAGGAAAGATGAAGGTTGGTGCAAATCATATCCTGAAGTTGTTATCCTAACCCATTTAA

CATAACATTTTACAACGTTTCCCAAATTTTTCCCTACGAATTAAACTTCTGCATTTCTAAATTTTTATACTATCTATAAATATAGACTTACAAGATGTGAGTGACTTACTTGGCACCGGTTCCCTGCCCTAGGTGGAGTTGAGTAGTTACTCAGATTTT

CCAACACATTATCAATAATCTGGTCATTGCTTTCACGCGTTGGAGAGAGAATATTTTCAGGGAGAACTCTACTTTCGACAACACCTATCACTTGCCCAGGCGTTGTATTTACCCAATCATCTCTACTTTCCACCCCATTGGCGAAATCGGCCGGCG

TATTATTTTGACAGACTTCTTTATGTACCACTTCATTAGTAACTGGTATGGCGTCTGGATCTTCAACTGCTGCTTGCGTACTAGCTTTGGAAATGTACGAAAGGATTTCCGTCTTAAAACTCTGCAGAATTGTATCCACACTACCAACAACTGAAGC

CTGATACGAGAGAGCATTGGAAGACACCTCCTTCACGGACGCTACAGCAAAATCGATTTTTTGCTCCAGTCTATGAATCTCAGGTGCCAAAATTGAGTGAACAAGAGCTTTGATTTTTGATTCCTGACTGAGGTCTATAGGTGCCTCATTCGTCT

TCTGTCTAACTTCTTTTCTTTTCCCTCCTTCTTTCAACTTCTCACGCATCTTCTCTACATCCACTTTAGTCAGACCACCTCTAAACATCTCTTTACGGATAACAACGTTATTCAAAATAAGATTGACCAAGTTATCAACCTTGACATCAACAACATCA

TCTGACCAATTAACCAAAGAGTCGACGTCACGCCTTGGAGGATCAACTGGAAGAATGCTGGTCACCAATGCCTACGATACACAGTAAAAAAAAGTTATTAGTGCATATGGGGTGCCGTTACTTTATTTACTGATTACGAACATATACGGTAATAA

AAACACCTCAGCTTTCCTATCAATTTCGCGAGCATGTCCCGGACTTAATGTCTGCTTCTTTGTCTTTCCGGGTAAACGATCACCGTCCTCTTCATCGCTCTCAGACTCTGATGAAGAATCAGCTTCTTGTACGACTTCTGTGAGTGAAGGAACTGC

TTCAACGATAACAAGCTGGAGGGCGAGGGCAAATCCTTTCAAGGCTATTGTGTTTTGCGACAGTGAAATCTCATCTCTTTTTTTAATACTTGTCATCAACATATCAAAAGCTAGTCTCCCCCAAGGGAAGGAAAAAAAATCGTCCATATCCACCA

GCAGCTCAGCGTGTTCCTTCAACATCTTCATACGCAGATTTGTGCAGAGCAAAACTGAAGACACAATTGCAAGGCAAGCCAGTTTCATCCGGAGATCTTTGTCGGTAACGGTTTTCCTCCGCAGCATTTTTACCGCTCTAGATACTCGCATTTCT

TCTACCCTTCCGAATAACTCCGGCCAATACGGTTTCTCCTTCATATTTTTCTTCTTCTTCGACTTTGATGTCTTTGGAAATTCACCACAAGGAAGACCGGTCACTATGGCAAACTCCCGTAGCGAGAATCTAATTGGTTTACCAGCGAATCGAAAC

CAAGCCTCGTGCTTTTTATCAATTTTAAGTTGCCTTGACAATAGATATCTAGCAAATCTACCAGAAAAAGCTGGTTTTTCCGAAATTTCGACCAACTTTCCGAATGAAGTCCCTTTCAGCCGCTCGATCTCGTCCGCATCTAGAGCTTCAAGAATT

GTGTTTAGCGCCCGCGACGACTGGTATGTCAGAACCCTAACACCGACGGGTTCCTCTCCCTCGGCGAACATCATTTCCGGTAAGGATACGGTGGTTTCCTCCATTTCTTCTTCCGCGAAATCACGACTCCGTGAAGAGGCCATCTCGATTACGAA

AACTGTGTGCTTGATAGAGGAGTGAAAATAAAACGCCCGATGAGAGATGAAAGAAAATGAAAGGCGTATAATACAAAGGAATCGACTAATATACTATAAAAAGGGATAGATATAAGGCAAGATAGGTTTTTTGCACGCTTTGAAAGTACATTTG

CATACTGTACCAAAGATATTTTCTACTACAGTACAAAGCGTGTTCTACCGCCTTCTCCCGGTTGTCTGCACAAAGCGCCATCCTAGTTTTGGGGACCTACATCCTCCGTTCTCTCTCATACTGAGGGTACTGTCGGTATTACCGAAAACTCAACAG

TACGATGCCCATCCACCTAATTTACATCTCTTCCATGTATATCTAGTGCAAAAACCC

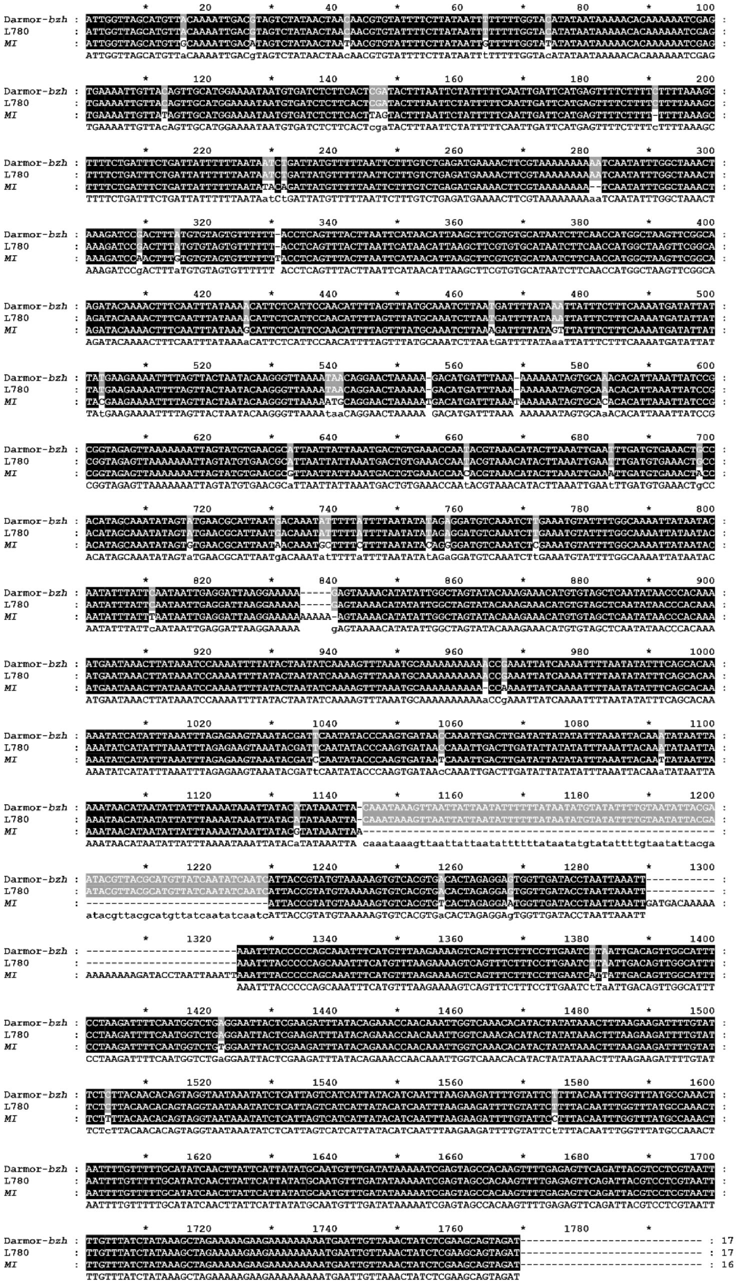

Figure S7. The promoter region sequence alignment of *BnaA05g08910D*. The chart contains 4 sequences, the gene sequence of Darmor-*bzh*(as a reference), the gene sequence of L780 and *MI* , the consensus line. Spaces indicate insertion or deletion and common bases are in black.

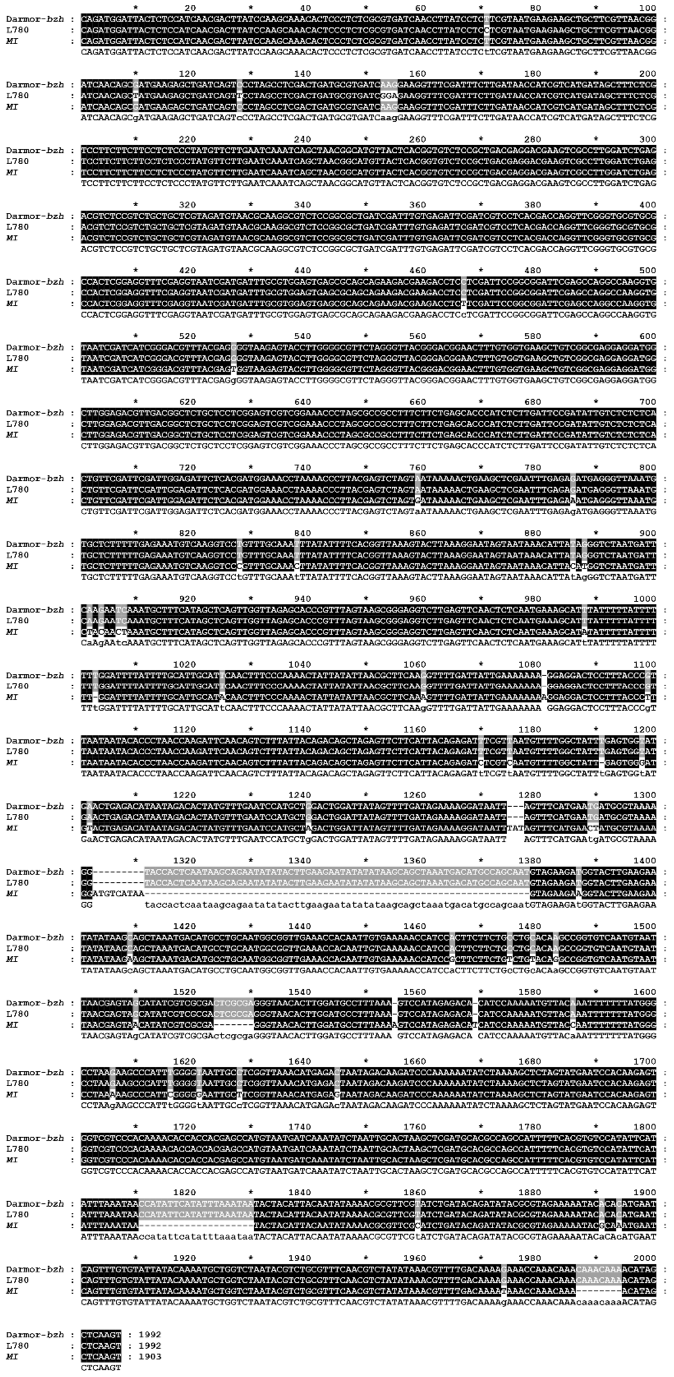

Figure S8. The promoter region sequence alignment of *BnaA05g08950D*. The chart contains 4 sequences, the gene sequence of Darmor-*bzh*(as a reference), the gene sequence of L780 and *MI* , the consensus line. Spaces indicate insertion or deletion and common bases are in black.
