# Supplementary material for: Identification and Fine Mapping of the Candidate Gene Controlling Multi-Inflorescence in Brassica napus
Source: Int J Mol Sci. 2022 Jun 29;23(13):7244. doi: 10.3390/ijms23137244 (PMC9266383; doi:10.3390/ijms23137244)
Supplement: Supplementary file 1 [file ijms-23-07244-s001.zip › Supplementary File S3.pdf]

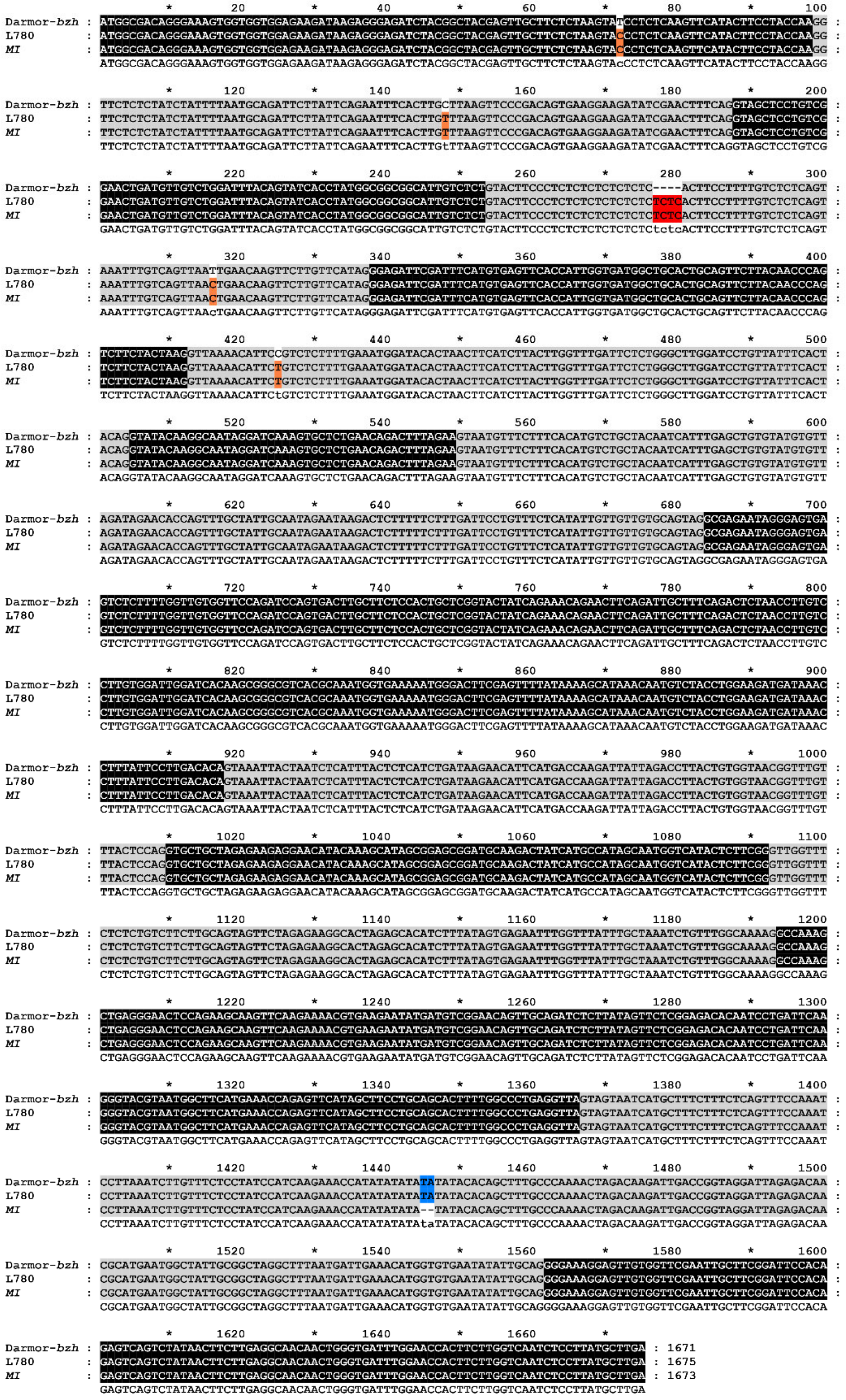

Figure S9. The nucleotide sequence alignment of *BnaA05g08870D*. The chart contains 4 sequences, the gene sequence of Darmor-*bzh*(as a reference), the gene sequence of L780 and *MI* , the consensus line. Orange represents SNP, red represents insertion, and blue represents deletion; exons are in black and introns in darkgray.

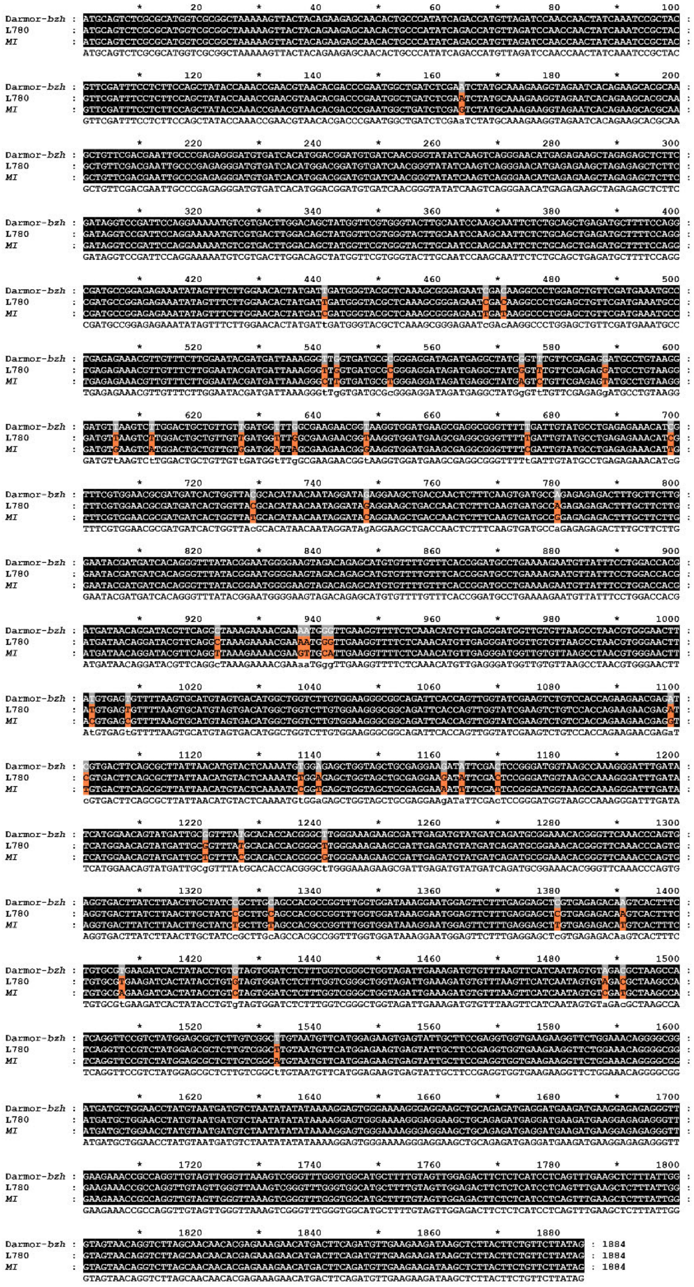

Figure S10. The nucleotide sequence alignment of *BnaA05g08880D*. The chart contains 4 sequences, the gene sequence of Darmor-*bzh*(as a reference), the gene sequence of L780 and *MI* , the consensus line. Orange represents SNP; exons are in black.

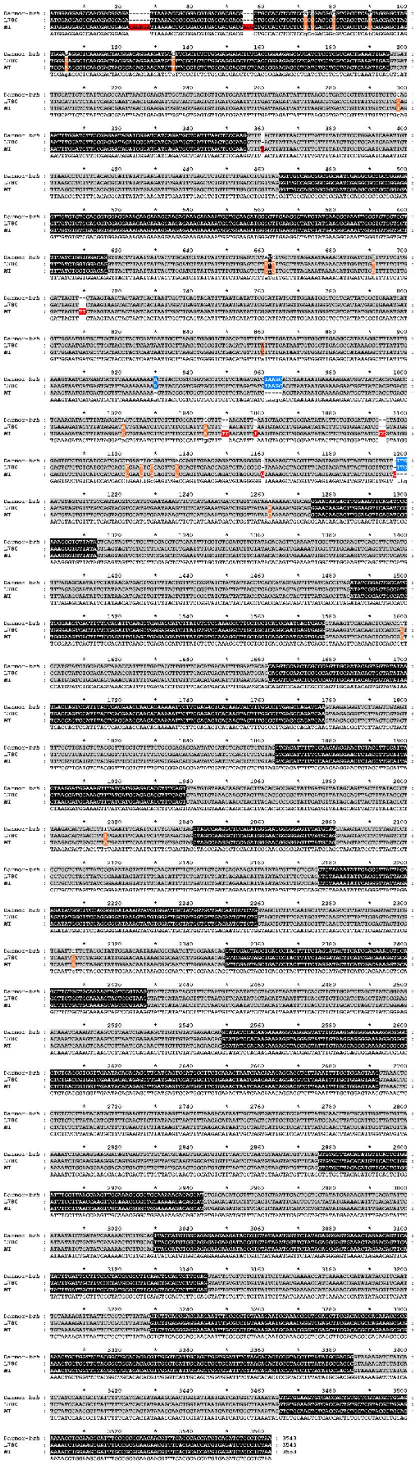

Figure S11. The nucleotide sequence alignment of *BnaA05g08890D*. The chart contains 4 sequences, the gene sequence of Darmor-*bzh*(as a reference), the gene sequence of L780 and *MI* , the consensus line. Orange represents SNP, red represents insertion, and blue represents deletion; exons are in black and introns in darkgray.

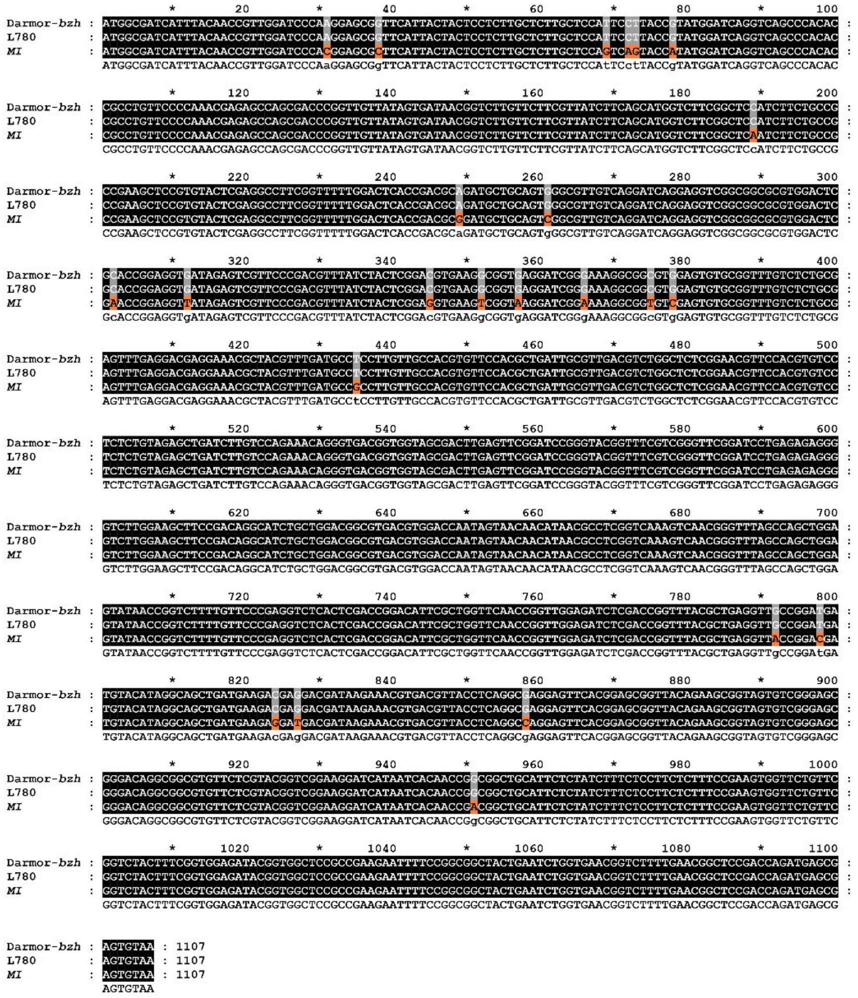

Figure S12. The nucleotide sequence alignment of *BnaA05g08900D*. The chart contains 4 sequences, the gene sequence of Darmor-*bzh*(as a reference), the gene sequence of L780 and *MI* , the consensus line. Orange represents SNP and exons are in black.

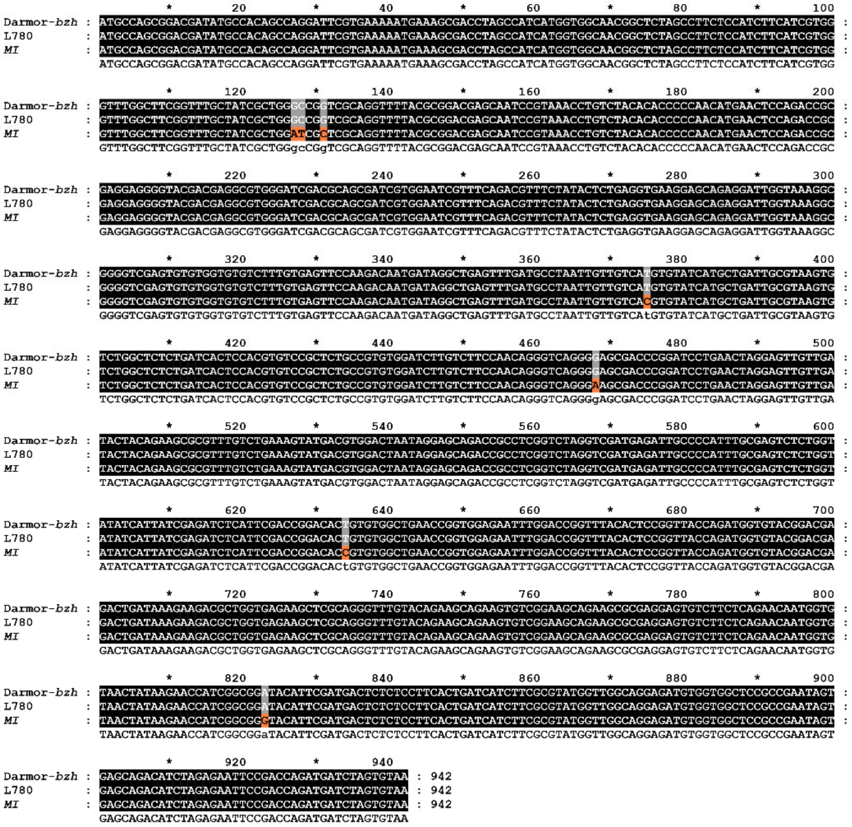

Figure S13. The nucleotide sequence alignment of *BnaA05g08910D*. The chart contains 4 sequences, the gene sequence of Darmor-*bzh*(as a reference), the gene sequence of L780 and *MI* , the consensus line. Orange represents SNP and exons are in black.

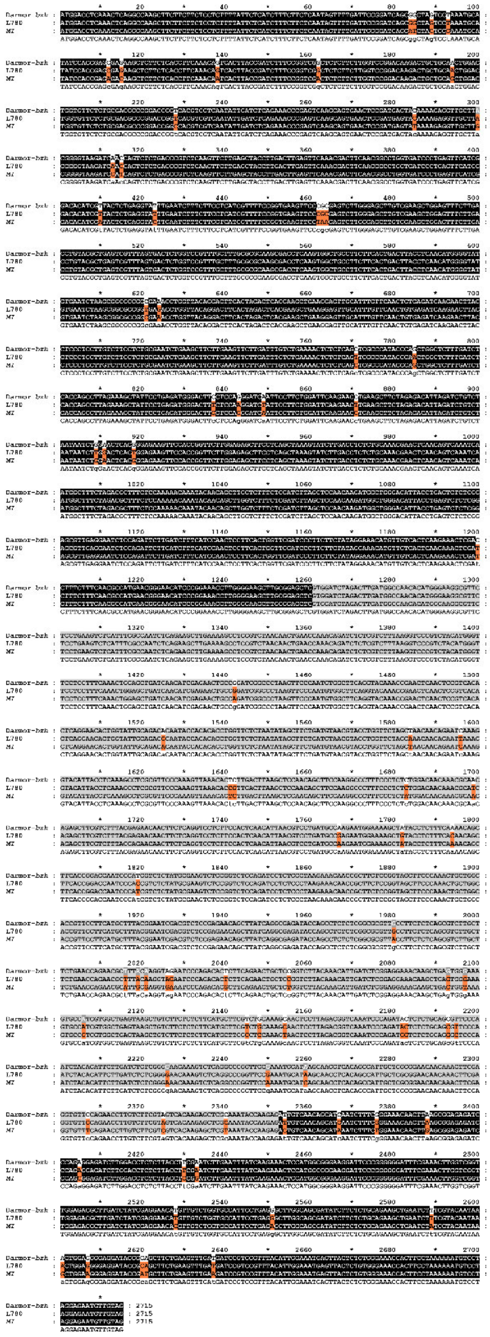

Figure S14. The nucleotide sequence alignment of *BnaA05g08950D*. The chart contains 4 sequences, the gene sequence of Darmor-*bzh*(as a reference), the gene sequence of L780 and *MI* , the consensus line. Orange represents SNP; exons are in black and introns in darkgray.
